# The Biological Macromolecule Crystallization Database and NASA Protein Crystal Growth Archive

**DOI:** 10.6028/jres.101.032

**Published:** 1996

**Authors:** Gary L. Gilliland, Michael Tung, Jane Ladner

**Affiliations:** Center for Advanced Research in Biotechnology of the University of Maryland Biotechnology Institute and National Institute of Standards and Technology, 9600 Gudelsky Dr., Rockville, MD 20850, U.S.A.

**Keywords:** Biological Macromolecule Crystallization Database, crystal, microgravity, NASA PCG Archive, nucleic acid, peptide, protein, protein crystallization, x-ray diffraction

## Abstract

The NIST/NASA/CARB Biological Macromolecule Crystallization Database (BMCD), NIST Standard Reference Database 21, contains crystal data and crystallization conditions for biological macromolecules. The database entries include data abstracted from published crystallographic reports. Each entry consists of information describing the biological macromolecule crystallized and crystal data and the crystallization conditions for each crystal form. The BMCD serves as the NASA Protein Crystal Growth Archive in that it contains protocols and results of crystallization experiments undertaken in microgravity (space). These database entries report the results, whether successful or not, from NASA-sponsored protein crystal growth experiments in microgravity and from microgravity crystallization studies sponsored by other international organizations. The BMCD was designed as a tool to assist x-ray crystallographers in the development of protocols to crystallize biological macromolecules, those that have previously been crystallized, and those that have not been crystallized.

## 1. Introduction

**Fig. 1:**
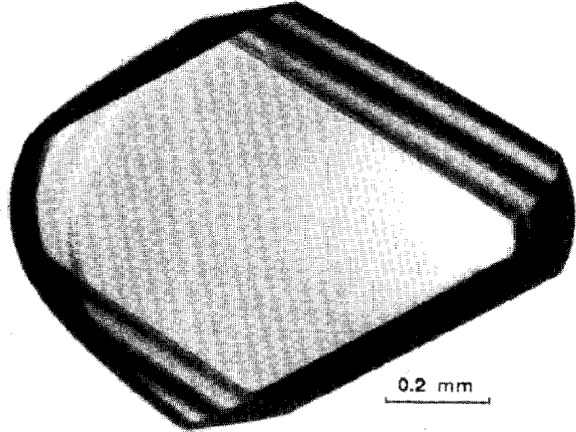
A photomicrograph of a crystal of bovine ribonuclease S grown aboard the Space Shuttle Flight STS-65 during the July 8th mission. The crystal was grown in a vapor diffusion reactor of the Advanced Protein Crystallization Facility at 20 °C. The droplet contained 25 mg/mL protein, 0.7 M ammonium sulfate, 1.8 M sodium chloride, and 0.05 M sodium acetate at pH 5.0. This was equilibrated against a reservoir solution composed of 1.4 M ammonium sulfate, 3.6 M sodium chloride, and 0.1 M sodium acetate, pH 5.0.

The three-dimensional structures of biological macromolecules have become essential elements for guiding studies and developing an understanding of biological processes. The coordinates provide essential information for protein engineering, rational drug design, and other biotechnology efforts. X-ray crystallographic studies of biological macromolecules provide a mechanism of attaining this information. The diffraction studies require the production of large single crystals of the biological macromolecule of interest. An example of a crystal of ribonuclease S suitable for crystallographic studies is shown in Fig 1.

The solution properties of a biological macromolecule, determined by factors such as size, shape, surface complexity, and conformational stability, directly relate to its ability to crystallize. A number of recent studies of the biophysical properties of biological macromolecules to discover simple methods that would have predictive capabilities have been prompted by recognition of these facts (i.e., Refs. [1–2]). Traditionally, empirical procedures consisting of a series of experiments that vary different parameters such as pH, temperature, ionic strength, and macromolecule concentration are carried out (for a recent review see Ref. [3]) to avoid the current lack of understanding of the crystal growth process needed to predict crystallization behavior. The number of experiments required for success is dependent on the macromolecule and the choices made by the investigator. If crystallization occurs over a broad range of conditions, or if the right choices are made in the initial experiments, the search ends quickly. In other cases many experiments are required to discover crystallization conditions, and occasionally no crystallization conditions are found, regardless of how many experiments are done.

Although several systematic procedures and strategy suggestions have been put forth [4–11], no *universal* strategy for searching for the crystal growth parameters for a biological macromolecule has gained widespread acceptance. Many laboratories have recently begun using experimental procedures called “fast screens” [12] that use sparse matrix sampling techniques [6]. These procedures employ a set of experiments that scan a wide range of pH and a variety of precipitants, buffers, and additives that have proven successful in other crystal growth studies. These strategies are based on the successes of many scientists, that have produced suitable crystals for diffraction studies for a variety of macromolecules.

The development of a productive crystallization strategy may yield crystals, but the internal order of a crystal dictates the quality of x-ray diffraction limiting the information that can be obtained from the ensuing crystallographic studies. An improvement in the diffraction quality of the crystals will not only facilitate the structural investigation, but also directly influence the accuracy of the coordinates obtained from the structural investigation. Recently, a number of crystallization experiments undertaken in space indicate microgravity has a positive influence on the crystal growth process, i.e., an improvement in the diffraction quality of crystals of biological macromolecules [13]. Because of these findings, several laboratories throughout the world are investigating the role of gravity in the crystallization process. Many additional crystallization experiments will be required to discover the reasons for the observed differences in the diffraction resolution observed between earth and space grown crystals.

The Biological Macromolecule Crystallization Database (BMCD), besides cataloging and summarizing the information concerning crystallization that is available in the literature, now includes the National Aeronautics and Space Administration (NASA) Protein Crystal Growth Archive. A description of the data and software that constitute the BMCD and the NASA Protein Crystal Growth Archive along with illustrations of how the BMCD can be used to address the needs of macromolecular crystallographers in the search for crystallization protocols is presented here.

## 2. The Biological Macromolecule Crystallization Database (BMCD)

In 1989, with assistance from the National Institute of Standards and Technology Standard Reference Data Program, the NIST/CARB (Center for Advanced Research in Biotechnology) BMCD database was created. The menu driven software used to access the data is written and compiled with Clipper^1^,^2^ and runs as an independent program on personal computers (PCs). All database files are in dBase III Plus^3^ format, and all of the index files are in the Clipper indexing format. The initial data consisted of the crystallization conditions of 1,025 crystal forms of 616 biological macromolecules. This information, which includes most of the crystallization protocols of biological macromolecules in the literature through the end of 1982, had been deposited earlier in the Brookhaven Protein Data Bank [14]^4^.

A second version of the NIST/CARB BMCD software and data was released in 1991 [10]. This release included data for 1,465 crystal forms of 924 biological macromolecules. In 1994 the BMCD became the NASA Protein Crystal Growth (PCG) Archive and began including data from crystal growth studies supported by NASA [15]. The software was expanded and released as the NIST/NASA/CARB BMCD Version 3.0 including data for 2,218 crystal forms of 1,465 biological macromolecules through 1994.

Recently, Version 3.0 of the BMCD has been ported to a UNIX platform to take advantage of the development of network capabilities that employ client-server tools [16]. This implementation of the BMCD uses the POSTGRES database management system [17] and World Wide Web/NCSA Mosaic client-server protocols [18]. The network version provides most of the features of the earlier PC versions of the BMCD. It also gives the user community access to the most recent updates, and it allows the rapid incorporation of new features and capabilities of the software.

## 3. BMCD Data

The BMCD includes crystallization data for peptides, proteins, protein-protein complexes, nucleic acids, nucleic acid-nucleic acid complexes, protein-nucleic acid complexes and viruses for which diffraction quality crystals have been obtained and reported in the literature. In addition, the BMCD, serving as the NASA Protein Crystal Growth Archive, contains the crystallization data generated from ground-based and microgravity crystallization studies supported by NASA. Crystallization data from microgravity experiments sponsored by other international space agencies are also included.

The information contained in the BMCD is divided into three major categories, biological macromolecule, crystallization, and summary data. Each biological macromolecule included in the database has a unique biopolymer sequence. For example, a mutant protein with a single amino acid change from the wild type protein is considered a separate macromolecule entry. Analogously, a crystal reported in the literature must have undergone a preliminary x-ray crystallographic study and have unique unit cell constants. Crystal entries that are nearly isomorphous with previously reported crystals are included if the author(s) describes significant differences in the intensity distribution of the diffraction pattern from that previously reported. The BMCD data elements for each category mentioned above are described below. Many data items, which are not appropriate for the biological macromolecule or crystal form or which may be missing from the published report, are not included in the data entries.

### 3.1 Biological Macromolecule Entry

**Fig. 2:**
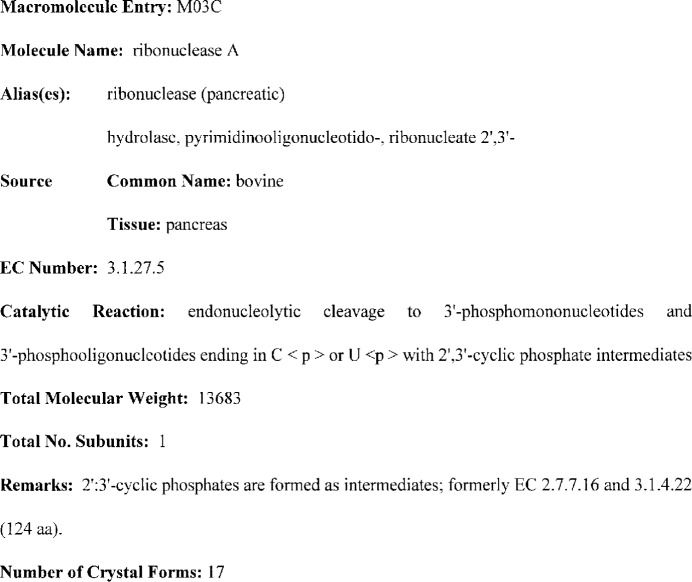
A representative example of the biological macromolecule entry M03C for ribonuclease A in the BMCD.

The entries for each biological macromolecule are composed of data elements that provide a unique description of the biopolymer or biopolymer complex. The data elements include the preferred name, the systematic name, and other aliases of the macromolecule. Each entry contains biological source information that consists of the common name, genus-species, tissue, cell, and organelle from which the macromolecule was isolated. Attempts have also been made to include this information for recombinant proteins expressed in a foreign host. The subunit composition and molecular weight are present. This information consists of the total number of subunits and the total molecular weight, and the number and corresponding molecular weights of each type of distinct subunit associated with the macromolecule. A subunit of a biological macromolecule is defined as a part of the *in vivo* assembly associated with other parts by noncovalent interactions. For example, the two monomers of a dimeric enzyme and the two oligomeric nucleic acid strands of a double-stranded nucleic acid fragment are considered in both cases as two subunits. The name of a prosthetic group associated with a biological macromolecule is included if it is reported in the crystallographic studies. If the macromolecule is an enzyme, the EC number [19] and catalytic reaction are provided. Finally, general remarks are included for cases where special features of the macromolecule are important, or might influence, its crystallization behavior. A four character alphanumeric identifier beginning with the letter M is assigned to each biological macromolecule entry. A macromolecule entry for an enzyme, bovine ribonuclease A, is illustrated in Fig. 2.

### 3.2 Crystal Entry

**Fig. 3:**
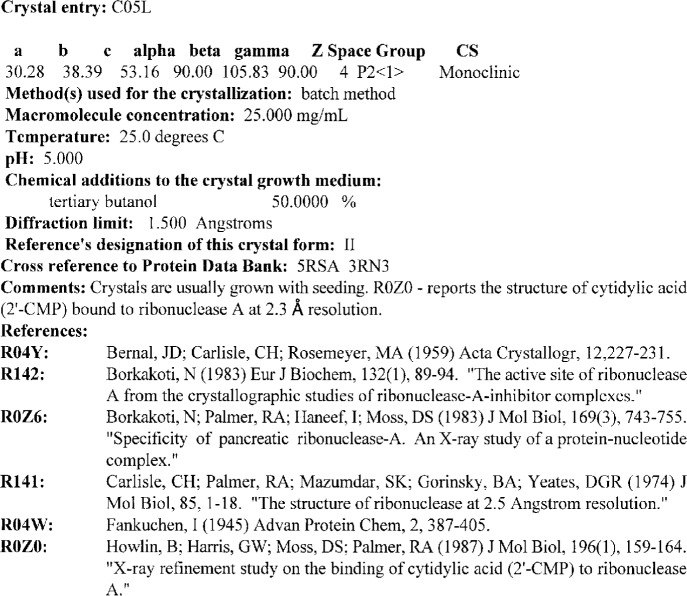
The BMCD cyrstal entry C05L, a representative example of one of the 17 crystal forms on ribonuclease A macromolecule entry M03C in the BMCD. It should be noted that the concentrations of chemical additives use four decimal places to accommodate the range of values of concentrations of the database, not to indicate the precision of the data.

Each crystal form of a particular biological macromolecule characterized by diffraction studies is described as a crystal entry. The data in each crystal entry includes the crystallization procedure, the crystal data, crystal morphology, and complete references. All of the details required to reproduce the crystallization procedure are data elements of the crystal entry. These elements consist of the crystallization method, the macromolecule concentration, the temperature, the pH, the chemical additives to the growth medium (buffer, precipitant and/or stabilizers), and the length of time required to produce crystals of a size suitable for diffraction experiments. If the crystallization deviates from standard protocols [7], an outline of the procedure is provided in the comments. The crystal size and shape are given along with the resolution limit of x-ray diffraction as described in the reference. References are provided for published crystal photographs or diffraction pictures. The crystal data include the unit cell dimensions (*a*, *b*, *c*, *α*, *β*, *γ*), the space group, the number of molecules in the unit cell (*Z*), and the crystal density. Data entries are also cross referenced to other structural biology databases such as the Brookhaven Protein Data Bank [14]. Each crystal entry is also given a four character alphanumeric identifier beginning with C. A crystal entry for the macromolecule entry illustrated in Fig. 2 is shown in Fig. 3.

### 3.3 Summary Information

The summary information gives the user a convenient mechanism for browsing the BMCD data. The user has access to a complete list of macromolecule names, tabulations of the number of macromolecules and crystal forms for each source, prosthetic group, space group, chemical addition, crystallization method, and a listing of complete references. The panels in Figs. 4a and 4b illustrate part of the summary information for the chemical addition and crystallization method tabulations.

Facilities are provided for comprehensive searches of the reference information. References can be queried for matches with a particular author, a key word or phrase. The BMCD also provides a listing of general references concerning all aspects of crystal growth. These references have been divided into categories that include reviews and books, articles concerning procedures, and references concerning nomenclature. Most references include complete titles, and remarks are often added to emphasize important aspects of a reference that may not be evident from the title.

## 4. The NASA PCG Archive

**Fig. 5:**
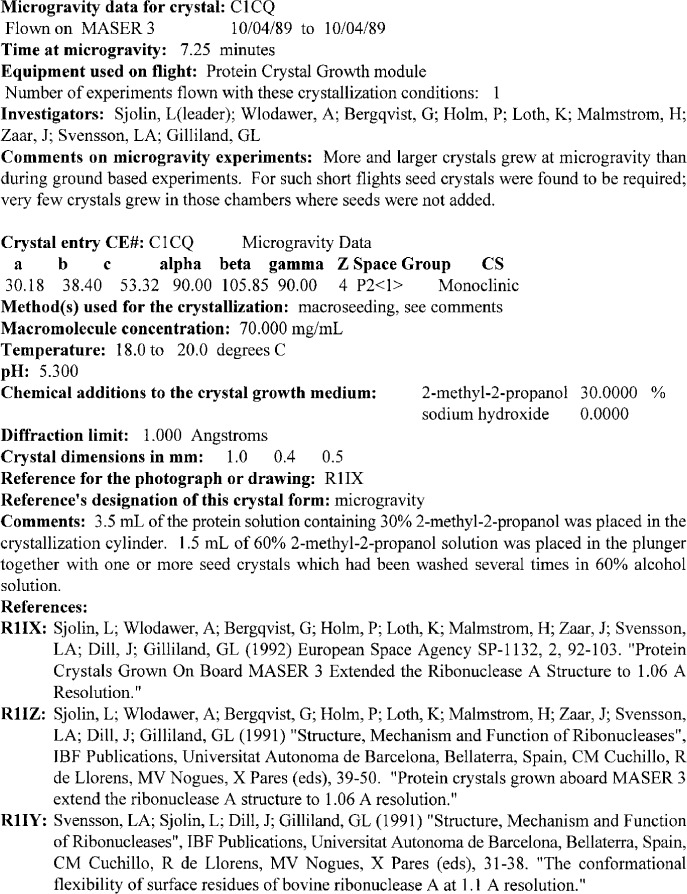
A representative example for a microgravity crystallization entry (C1CQ) of ribonuclease A (M03C) in the BMCD.

As briefly mentioned above, a new function of the BMCD is to serve as the NASA PCG Archive. A complete description of the biological macromolecule crystallization experiments performed in microgravity in the NASA Space Shuttle and in ground control experiments is included in the BMCD. In addition, the BMCD includes data and results from microgravity crystallization experiments sponsored by other international space agencies. The data are organized in a similar way as other entries in the BMCD. The data comprising the PCG Archive mentioned above contains biological macromolecule and crystal entries as described above with additional data items that relate to the microgravity experiment(s). In fact, in the PCG Archive a single crystal form of a macromolecule may have multiple entries, one for each microgravity experiment. Each of these crystallization entries is also given a four character alphanumeric identifier beginning with the letter C. An example of a microgravity crystal entry for a ribonuclease A (entry M03C, Fig. 2) experiment is shown in Fig. 5.

As part of the NASA archive, new summary displays of information specific to the microgravity crystallization experiments have been added to the BMCD. Included are lists of the apparatuses used in microgravity experiments, of the microgravity missions, of the acronyms and names of sponsors, and of references discussing theory or experimental results of crystallization of biological macromolecules in microgravity. Detailed descriptions of the crystallization apparatuses that are in use for microgravity crystallization experiments and that have grown crystals are also available.

## 5. Querying the BMCD

Both the PC and WWW versions of the BMCD provide user interfaces for querying the database. Database searches may be carried out for data elements of any of the categories listed in Table 1. Depending upon the element selected, a parameter value (numerical or text) or a range of values (numerical) is requested. The interfaces provide mechanisms that allow the use of the Boolean logic **AND** and **OR** functions to generate quite complex searches. The results of searches can be displayed in a variety of ways and can be written to an ASCII file or printed.

## 6. Crystallization Strategies

From its inception the BMCD has been designed as a tool to assist in the development of crystallization strategies for biological macromolecules. These strategies range from reproducing published procedures to crystallizing a biological macromolecule that is unrelated to any that has previously been crystallized [10]. Discussed below are four general categories of problems that present themselves. These include developing strategies for the crystallization (1) of a previously crystallized biological macromolecule, (2) of a modified or mutant biological macromolecule for which the unmodified or wild-type biological macromolecule has been reported, (3) of a biological macromolecule that is homologous to previously crystallized macromolecule(s), and (4) of a biological macromolecule that was not previously crystallized.

### 6.1 Previously Crystallized Macromolecules

The BMCD contains the information to reproduce the crystallization conditions for biological macromolecules reported in the literature. The reported crystallization conditions are the starting points to initiate the crystallization trials. The crystallization of the biological macromolecule may be quite routine, but differences in the isolation and purification procedures, reagents, and crystallization methodology of laboratories can influence dramatically the reproducibility, and therefore, the successful outcome. The crystallization conditions in the database should be considered as an initial starting point that may require experiments that vary pH, macromolecule and reagent concentrations, temperature, along with the crystallization method. The protocol used to produce crystals for the structure determination of bovine chymosin [20] represent the adaptation of a procedure published many years earlier [21]. The modified procedure used the hanging drop vapor diffusion procedure [22] that was not being practiced when the first crystal growth studies were reported.

### 6.2 Variant Macromolecule or Ligand-Biological Macromolecule Complexes

The structures of sequence variants, chemically modified macromolecules, or ligand-biological macromolecule complexes are becoming routine structure determination problems for crystallographic laboratories. Frequently the altered biopolymer sequence, chemical modification, and presence of the ligand do not interfere with crystal packing interactions, significantly alter the conformation, or influence the solution properties of the macromolecule. If this is the case, the crystallization conditions of the biological macromolecule may be similar to the wild type, unmodified or the “free” biological macromolecule. This may be true for even quite radically modified variants such as the nine residue, high-affinity calcium site deletion mutant of subtilisin BPN’ [23]. This variant crystallized under virtually identical conditions with that of the wild type molecule.

### 6.3 Homologous Biological Macromolecules

Frequently, crystallographic studies are initiated with a biological macromolecule related through sequence (and presumably structural) homology to a set of macromolecules that have previously been crystallized. The BMCD can be used to tabulate the crystallization conditions for the members of the family, and then this information can be used to limit the parameters for the initial crystallization trials with the biological macromolecule. For example an examination of crystallization conditions for the single-chain acid proteinases listed in Table 2 indicate that most crystals are grown using a variety of the precipitants: ammonium sulfate, lithium sulfate, ethanol, polyethylene glycol 4000, and polyethylene glycol 8000. One half of the crystal forms listed in Table 2 utilize ammonium sulfate suggesting that this would be the reagent to use in the initial trials. The protein concentration ranges from 2 mg/mL to 50 mg/mL, the pH ranges from 1.5 to 6.3, and the temperature ranges from 4 °C to 26 °C. These reagents and parameters would then represent a logical starting point for initiating a limited set of crystallization trials. If no crystals were obtained using this information, the more general procedure outlined next would be warranted.

### 6.4 General Crystallization Procedure

**Fig. 6:**
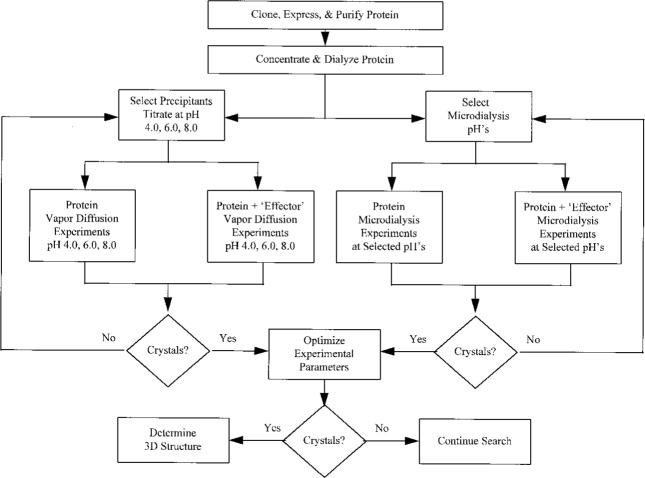
A general strategy for finding the crystallization conditions for a recombinant protein based on the data in the BMCD.

The use of the BMCD has been incorporated into general procedures required for the crystallization of a unique biological macromolecule that has never been crystallized [9, 10]. A general procedure for a soluble recombinant protein is illustrated in Fig. 6. Once a protein is cloned, expressed, and purified, it is concentrated (if possible) to at least 10 mg/mL, and preferably to a concentration of at least 25 mg/mL or higher. It is then dialyzed into 0.005 to 0.025 M buffer at neutral pH or at a pH required to maintain solubility of the protein. Other stabilizing agents such as EDTA and dithiothreitol may be included at low concentrations to stabilize the biological macromolecule during the crystallization trials. Once this has been accomplished, two different types of experimental procedures are carried out concurrently, one employing the vapor diffusion method and the other microdialysis.

Vapor diffusion experiments are set up after selecting from 3 to 10 of the reagents that have been most successful at inducing crystallization (Fig. 4a) for soluble proteins. The data in the BMCD is also used to set the limits for parameters such as pH and temperature. A distribution of the number of crystal forms for all biological macromolecules versus pH and temperature is shown in Figs. 7a and 7b, respectively. For the procedure illustrated in Fig. 6, three different pH’s are selected, 4.0, 6.0 and 8.0 and experiments are done at both room and coldroom temperatures, approximately 20 °C and 6 °C, respectively. Before actually setting up the vapor diffusion experiments, small aliquots of concentrated buffered reagents (0.1 M buffer) are added to a small droplet (10 μL) of the protein in a stepwise manner to determine the protein’s precipitation point [4]. After each addition of 0.5 μL to 1.0 μL of the reagent, the droplet is sealed in an airtight chamber and observed after 10 min. If precipitation occurs, distilled water can be added to the droplet to attempt to redissolve the protein. These titration experiments set the limits of reagent concentrations for the vapor diffusion experiments. These should be done at both room and coldroom temperatures.

Once the precipitation points have been found, sets of vapor diffusion experiments covering the range of reagents and pH conditions are initiated at both room and coldroom temperatures. Small droplets of protein (5 μL) are mixed with an equal volume of a reservoir solution and placed in a sealed chamber that allows the droplet to equilibrate with 0.5 mL to 1.0 mL of the reservoir solution. At the selected pH, successive experiments are initiated that increase the precipitant concentration to a value greater than that observed in the titration experiment. For example, if a protein precipitates at 50 % saturated ammonium sulfate, experiments that equilibrate droplets against 44 %, 47 %, 50 %, 53 %, and 55 % saturated ammonium sulfate. An equivalent set of vapor diffusion experiments with protein solution containing an effector (a ligand, cofactor, metal ion, etc.) can be carried out in parallel.

Besides the precipitant (or crystallizing) reagents mentioned above, an analysis of the data contained in the BMCD reveals that about 10 % of the soluble proteins crystallize at low ionic strength (< 0.2 M). Thus, the procedure outlined in Fig. 6 indicates that a set of microdialysis experiments equilibrating the protein solutions against low ionic strength should be carried out for an unknown protein. Initially, experiments over a pH range of 3.0 to 9.0 in steps of 0.5 to 1.0 should be carried out, again, at both room and coldroom temperatures. It is also worthwhile to do microdialysis experiments at or near the protein’s isoelectric point, a point at which a protein is often the least soluble. As with the vapor diffusion experiments mentioned above, the introduction of small quantities of ligands, products, substrate, substrate analogs, monovalent or divalent cations, organic reagents, etc., to the crystallization mixtures may facilitate crystal growth. Thus, it is recommended that a second set of experiments be run in parallel to those described above.

After initiating the experiments above periodic assessment of the experiments is done. The time for crystals to appear ranges from a few hours to many months, but usually if there is no success within three weeks, further crystallization trials are suggested. For the vapor diffusion experiments, additional reagents should be selected from the BMCD summary and the titration carried out before setting up a new set of experiments. Alternatively, a finer increment in pH and/or other temperatures should be considered. For the microdialysis experiments additional experiments at a finer increment should be carried out. For both the vapor diffusion and microdialysis experiments other effectors or additives should also be considered.

If crystals are discovered in either the vapor diffusion or the microdialysis experiments, the parameters that are influencing crystal growth should be optimized. This requires small perturbations in the pH, temperature, and the reagent and protein concentrations. At this point seeding experiments should also be considered to improve crystal size or reproducibility. Even if crystals are large enough for diffraction studies, optimization may be necessary to improve the diffraction quality. If the crystals diffract well, the three-dimensional structure determination can be initiated, but if the crystals do not diffract, or diffract poorly, the search should be continued as mentioned above.

## 7. Future Developments of the BMCD

The primary goal of the BMCD will continue to be a complete, error-free, and up-to-date crystallization database and NASA PCG Archive. The availability of the BMCD on the Internet will simplify user reporting of errors and distribution of new data. The current version of the software is being expanded to include alternate ways for users to display the data. Because of the BMCD being one of several biological and crystallographic databases on the internet, direct links to related entries in other databases are being considered. Finally, software tools for users engaged in crystal growth studies are being developed that will assist the user in deciding what experiments are appropriate and in designing the experiments for a particular biological macromolecule. These tools incorporate the principles outlined in the discussion of crystallization strategies outlined above.

## Figures and Tables

**Fig. 4a:**
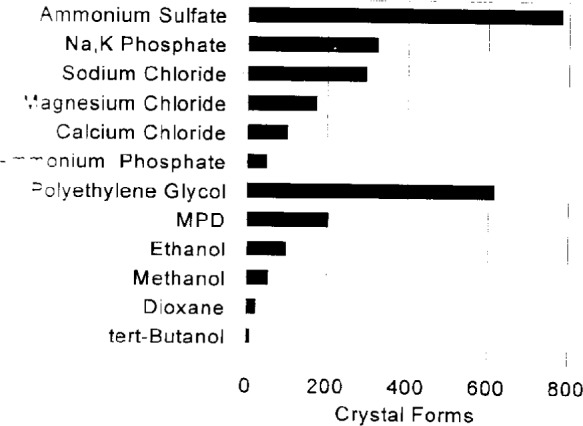
A display of the BMCD summary information for chemical additions to the crystallization solutions.

**Fig. 4b:**
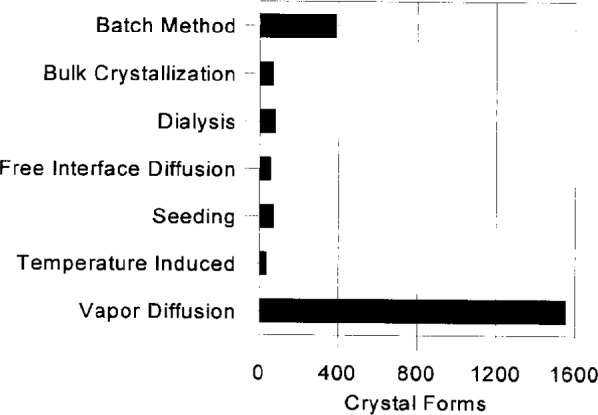
A display of the BMCD summary information for crystallization methods.

**Fig. 7a:**
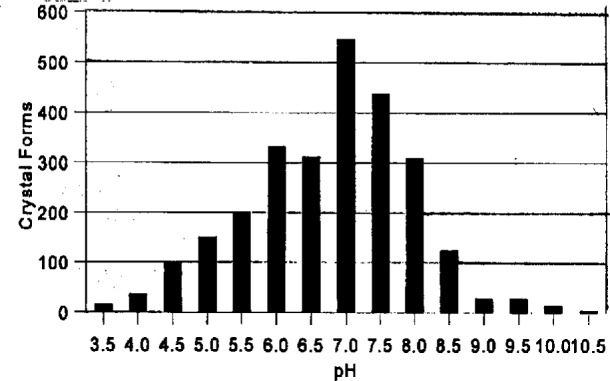
The distribution of the number of crystal forms in the BMCD as a function of pH.

**Fig. 7b:**
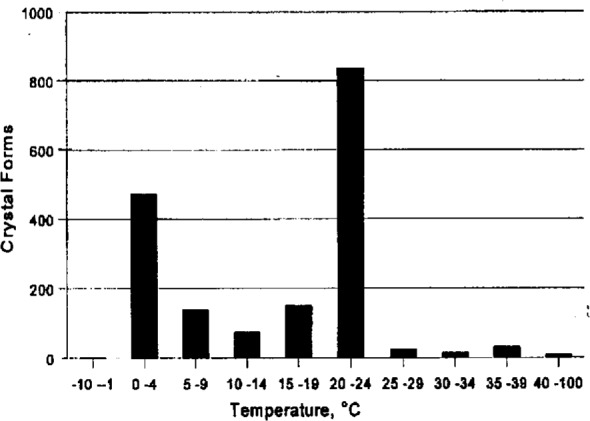
The distribution of the number of crystal forms in the BMCD as a function of temperature.

**Table 1:** The BMCD search parameters

**Macromolecule entry**		
Macromolecule name	Biological source	Molecular weight
Subunit composition	Prosthetic group	Multiple crystal forms
**Crystal entry**		
*Crystal data*	*Crystallization conditions*	*Reference*
Space group	Crystallization method	Author
Unit cell dimensions	Macromolecule concentration	Year
Z, molecules/unit cell	Crystallization Temperature	Journal
Crystal density	pH of crystallization Crystal growth time Chemical additions	Database ID

**Table 2:** The crystallization conditions of single-chain acid proteinases contained in Version 3.0 of the BMCD

Acid protease source	Crystal data (space group unit cell)	Crystallization method	Protein conc. (mg/mL)	Chemical additions^a^,^b^	pH	Temp. (°C)	Reference
Cathepsin D	P2_1_2_1_2_1_	vapor diffusion	12.5	21.0 % PEG 8000	4.0		[24]
Human	59.9, 99.6, 133.6			0.025 M sodium citrate			
				0.003 M potassium thiocyanate			
				0.005 M Tris-HCl			
	P6_1_	vapor diffusion	10.0	31.0 to 32.5 % ammonium sulfate	5.1	20	[25]
	125.9,125.9,104.1			0.025 M sodium acetate			
Chymosin	I222	vapor diffusion	8.6	2.0 M sodium chloride	5.0–6.2	20	[20]
Bovine, calf	72.8, 80.3, 114.8			0.05 M MES			
Cladosporium acid protease	P2_1_2_1_2_1_	vapor diffusion	20.0	16.0 % PEG 4000	5.2–6.0	4	[26]
Cladosporium	136.5, 109.4, 87.7			0.05 M cacodylate			
Endothiapepsin	P2_1_	batch	1.0–2.0	2.2 M ammonium sulfate	4.5–6.3	20	[27]
*Endothia parasitica*	53.5,74.2, 45.6, 109.1°			0.1 M sodium phosphate			
				0.25 % to 1.0 % acetone			
	P2_1_	batch	1.0–2.0	2.2 M ammonium sulfate	4.5	20	[27]
	55.6, 60.1, 45.8, 101.8°			0.1 M sodium phosphate			
	P2_1_	batch	2.0	2.2 M ammonium sulfate	4.6	22–26	[28]
+ inhibitor H261(1:10)	43.0, 75.7, 42.9, 97.1°			0.1 M sodium phosphate acetone			
Mucor acid protease	P2_1_2_1_2	batch	30.0	2.25 M ammonium sulfate	5.6	20	[29]
*Mucor pusillus v. Lind*	70.3, 104.3, 46.4			0.5 M sodium chloride			
				0.05 M phosphate			
				6.0 % acetone			
Penicillopepsin	C_2_	concentration	9.0	30 % ammonium sulfate	4.6	20	[30]
*Penicillium janthinellum*	97.8,46.7,65.7,115.9°			0.15 M sodium acetate			
Pepsin A	P2_1_	batch	20.0–25.0	20 % ethanol	1.5–2.0	20	[31]
Porcine	54.7,36.3,73.5,104.0°			0.1 M acetate			
Pepsinogen	C2	batch	30.0	52 % lithium sulfate	6.0	4	[32]
Porcine	106.1,43.7,88.9,91.4°			0.05 M sodium cacodylate			
Rhizopuspepsin	P2_1_2_1_2_1_	vapor diffusion	10.0–50.0	0.02 M calcium acetate	6.0	4	[33]
*Rhizopus chinensis*	60.3, 60.8, 107.1						

a Abbreviations: PEG, polyethylene glycol; Tris, Tris-(hydroxymethyl)aminomethane; MES, (2-(N-Morpholino)ethanesulfonic acid.

b Percents for PEG are weight/volume, for organic solvents are volume/volume, and for salts are percentage of saturation at the given temperature.

## Footnotes

1 Certain commercial equipment, instruments, or materials are identified in this paper to foster understanding. Such identification does not imply recommendation or endorsement by the National Institute of Standards and Technology, nor does it imply that the materials or equipment identified are necessarily the best available for the purpose.

2 Clipper is a registered trademark of Nantucket Corp., 12555 West Jefferson Boulevard, Los Angeles, CA 90066.

3 dBASE III Plus is a registered trademark of Borland International Inc.

4 Gilliland, unpublished data (1983).
